# Robust learning algorithms for capturing oceanic dynamics and transport of *Noctiluca* blooms using linear dynamical models

**DOI:** 10.1371/journal.pone.0218183

**Published:** 2019-06-13

**Authors:** Yan Yan, Tony Jebara, Ryan Abernathey, Joaquim Goes, Helga Gomes

**Affiliations:** 1 Data Science Institute, Columbia University, New York, NY, United States of America; 2 Department of Computer Sciences, Columbia University, New York, NY, United States of America; 3 Lamont Doherty Earth Observatory, Columbia University, Palisades, New York, NY, United States of America; Universidade de Vigo, SPAIN

## Abstract

The blooms of *Noctiluca* in the Gulf of Oman and the Arabian Sea have been intensifying in recent years, posing now a threat to regional fisheries and the long-term health of an ecosystem supporting a coastal population of nearly 120 million people. We present the results of a local-scale data analysis to investigate the onset and patterns of the *Noctiluca* blooms, which form annually during the winter monsoon in the Gulf of Oman and in the Arabian Sea. Our approach combines methods in physical and biological oceanography with machine learning techniques. In particular, we present a robust algorithm, the variable-length Linear Dynamic Systems (**vLDS**) model, that extracts the causal factors and latent dynamics at the local-scale along each individual drifter trajectory, and demonstrate its effectiveness by using it to generate predictive plots for all variables and test macroscopic scientific hypotheses. The vLDS model is a new algorithm specifically designed to analyze the irregular dataset from surface velocity drifters, in which the multivariate time series trajectories are having variable or unequal lengths. The test results provide local-scale statistical evidence to support and check the macroscopic physical and biological Oceanography hypotheses on the *Noctiluca* blooms; it also helps identify complementary local trajectory-scale dynamics that might not be visible or discoverable at the macroscopic scale. The vLDS model also exhibits a generalization capability (as a machine learning methodology) to investigate important causal factors and hidden dynamics associated with ocean biogeochemical processes and phenomena at the population-level and local trajectory-scale.

## Introduction

### Background

Recent advances in Data Science and Machine Learning have produced great successes in a variety of data-driven modeling for interdisciplinary scientific problems concerning complex natural phenomena, in a number of fields including Marine Ecology [1–6], Climatology [7], Oceanography [8–11], Geoscience [12], Computer Vision [13–15], Social Science [16], Computational Neuroscience [17–20], Speech and Language Processing [21–23], and Environmental Health Science [24]. Here we present a local drifter-scale data analysis technique to investigate the onset and patterns of the *Noctiluca* winter monsoon blooms, which form annually in the Gulf of Oman and in the Arabian Sea. Our approach relies on a combination of physical oceanography and machine learning techniques. In particular, we obtain a robust model, the variable-length Linear Dynamic System Model (vLDS mode, hereafter) that is capable of identifying the causal factors and dynamics at the local-scale population-level along each individual drifter trajectory. The difficulty of analyzing this dataset lies in its irregularity, in which all the multivariate time series trajectories do not share an equal length. This renders the conventional multivariate Linear Dynamical System (LDS) method unsuitable. The vLDS model is a new algorithm specifically designed to address this irregularity of the dataset. Furthermore, we assess the effectiveness of vLDS by generating predictive plots for all variables and testing macroscopic scientific hypotheses. Rigorously statistical, the vLDS model available in the supplementary materials (S1 Software) is a powerful tool that helps: 1) discover local trajectory-scale causal relationships in a high-dimensional dataset, 2) identify complementary local trajectory-scale dynamics that might not be discoverable at the macroscopic scale or accessible in controlled laboratory experiments, and 3) obtain a generalizable machine learning methodology to probe important local trajectory-scale causal factors and hidden dynamics for other trajectory-based datasets in marine ecology.

**Fig 1:**
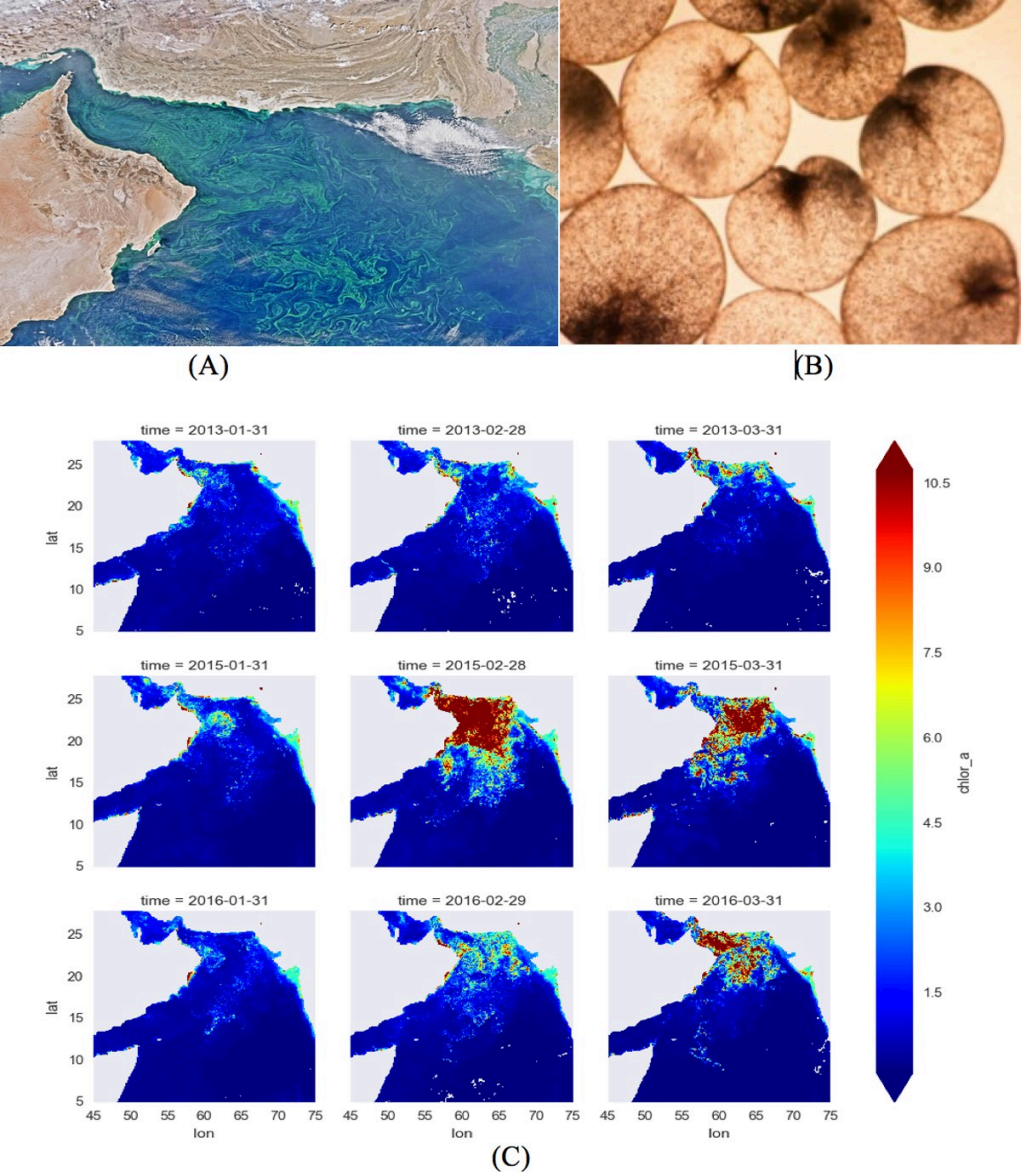
**(A)** Satellites images displaying *Noctiluca* blooms as large swirls on the surface of the Arabian Sea [40]. **(B)** *Noctiluca scintillans* with a flick of its tail-like flagellum drawing energy from the millions of green algae, or “endosymbionts”, captured inside its transparent cell walls. **(C)** Satellite image for the monthly data of the chlorophyll *a* concentration in the Arabian Sea during year 2013, 2015, and 2016.

The significance of this research is that these blooms of *Noctiluca* have been intensifying in recent years, posing now a threat to regional fisheries and the long-term health of an ecosystem supporting a coastal population of nearly 120 million people [25–28]. When seen from space, the *Noctiluca* blooms appear as large drifting swirls and filaments on the surface of the sea (Fig 1A and 1C). Traditionally, photosynthetic diatoms supported the Arabian Sea food chain. Zooplankton preyed on diatoms, a type of algae, and were in turn grazed by fish. The situation changed since the early 2000s, when researchers began to observe vast developments of *Noctiluca* blooms associated with a steep decline in diatoms. Within a decade, *Noctiluca* had virtually replaced diatoms at the base of the food chain, marking the start of a colossal ecosystem shift [26]. By previous macroscopic studies [25–39] based on satellite observations, ocean observations, in-*situ* data sampling, and biologically controlled experiments in the laboratory, a part of the underlying dynamics that governs the transport, growth and decay of the *Noctiluca scintillans* blooms in the Arabian Sea region has been disclosed. It has been demonstrated that *Noctiluca* can dive down with a flick of its tail-like flagellum, to eat plankton, living or dead, or swim up to the light, drawing energy from the millions of green algae, or “endosymbionts,” living within its transparent cell walls (Fig 1B). This flexibility gives it an edge on diatoms, which survive on sunlight alone. Putting *Noctiluca* and its diatom competitors in oxygen-starved water we found that *Noctiluca*’s carbon-fixation rate rose by up to 300 percent while the diatoms’ fell by nearly as much. S1 Appendix shows more details on the research development of the *Noctiluca* blooms in recent years.

### Goal and outline

To understand the local-scale impact of the physico-chemical and physical oceanographic factors at the population-level on *Noctiluca* blooms along drifter trajectories, we have collected, combined, and preprocessed data from both the Ocean and Satellite datasets. The trajectory of each drifter is recovered by its spatio-temporal information. The physical oceanographic profiles associated with the spatio-temporal coordinates of each drifter is then utilized to discern the behavior and movement of the *Noctiluca* blooms along the trajectory of the particular drifter. This behavior and movement is statistically learned or transformed by the vLDS model. Since the drifters have different launch time and longevity, the time series representing individual drifter trajectories have unequal (variable) lengths. To our knowledge, no existing model can simultaneously process regularly-sampled multiple multivariate time series with variable lengths collected by velocity drifters. The goal in this paper is to describe a variable-length Linear Dynamic Systems (vLDS) model that is tailored to this particular data structure, to learn, summarize, and recover the latent dynamics for all drifter trajectories, and to generate predictive dynamics that match closely with the observed data along drifter trajectories.

The previous analysis of the phytoplankton blooms in [25–26, 41–42] was based on the macroscopic scale of space and time, namely, the data is aggregated or pooled across spatio-temporal dimensions. This research hypothesized (1) that nutrient-enrichment of the surface waters are increasing productivity. The potential sources of nutrients are multiple [25–26]. Moreover, these previous research hypothesized (2) that *Noctiluca* grew faster in light than in dark on the sea surface and in the sea water, thanks to its sun-loving endosymbiotic algae, which are thought to have survived 1.3 billion years on an oxygen-scarce Earth. However, in our study, the surface velocity drifter dataset [43] and satellite image dataset [44–47] are not aggregated or pooled across spatio-temporal dimensions. With the data structure of individual drifter trajectories kept intact, the vLDS model tests the previous hypotheses on the dynamics of the *Noctiluca* blooms from the spatio-temporal trajectory-scale. By comparing the vLDS model predictions directly with the observed ocean profiles along the drifter trajectories, we can easily visualize its predictive performance and interpret the underlying latent dynamics of the *Noctiluca* blooms at the trajectory scale, as discussed in the “Discussion & conclusion” Section.

The benefits of vLDS are threefold. First, it provides statistical evidence in a direct and zoomed-in manner to compare the macroscopic physical and biological oceanographic observations and the inherent physiological behavior of *Noctiluca* blooms, by recovering the latent dynamics that governs the probabilistic distribution of the *Noctiluca* concentration in space and time and by comparing the model predictions with the observed ocean profiles. Second and more importantly, it helps identify complementary local drifter-scale dynamics that might not be visible or discoverable at the macroscopic scale. The vLDS predictive plots in “Discussion & Conclusion” Section provide statistical evidence that the atmospheric deposition measured by the quantities *T865* aerosol optical thickness at wavelength 865 nm does not have much impact on the underlying dynamics that are driving the *Noctiluca* growth, as measured by the chlorophyll *a* concentration (*Chl a*) at the time scale of two days. Third, it provides a generalizable machine learning methodology to probe important causal factors and hidden dynamics for the ocean biogeochemical processes at the local population-level along individual drifter trajectories. These scientific findings in the local trajectory-scale of the population data can lead to critical hypothesis and even conclusions at the macroscopic scale of the pooled data.

## Materials and methods

### Data collection

**Fig 2:**
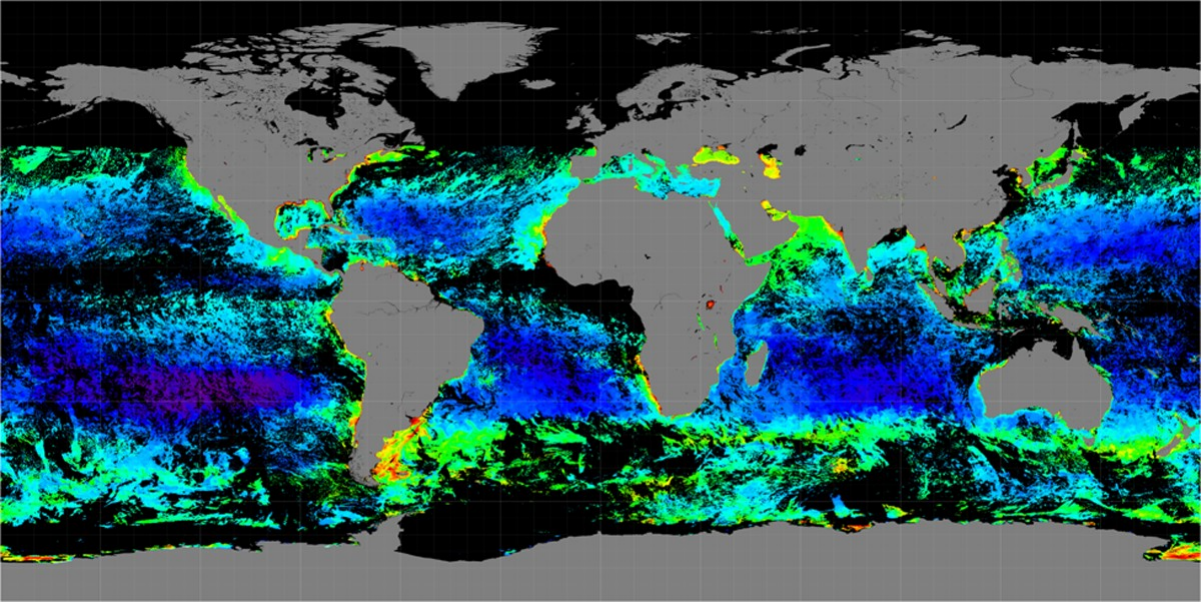
Level-3 data for the chlorophyll *a* concentration from Dec. 27 to Dec. 31, 2015 [40]. Dark regions indicate missing values.

The Arabian Sea (coordinate range ~5 to 28°N, 45 to 75°E) is predominantly located in the tropics (Fig 1A), and it has one of the most energetic current systems driven by the seasonally reversing monsoons. Dataset on Chlorophyll a (*Chl a*), from the GlobColour Project [45–47], which provides merged products based on measurements from the ocean color satellites Sea-WiFS, MODIS-Aqua (NASA), VIIRS (NOAA) and MERIS and OLCI-A (ESA), was used for studying the distribution of *Noctiluca* blooms during winter. In practice, the Ocean color datasets have missing values at certain locations due to the limitations of the satellite coverage or the presence of clouds. The dark spots are regions with missing values (Fig 2). For the purpose of our study, we used merged products from both NASA and the GlobColour Project [44–47]. Ocean color satellites can provide remote sensing reflectance values for different wavebands. These wavebands are used in empirical and semi-analytical algorithms to convert remote sensing reflectance to chlorophyll *a* concentration. Pre-processed *Chl a* data products were used to explore a time series of snapshots of chlorophyll *a* concentration on a lattice of latitude and longitude coordinates. In the next sections, we provide detailed descriptions of the data aggregation and preprocessing steps.

**Fig 3:**
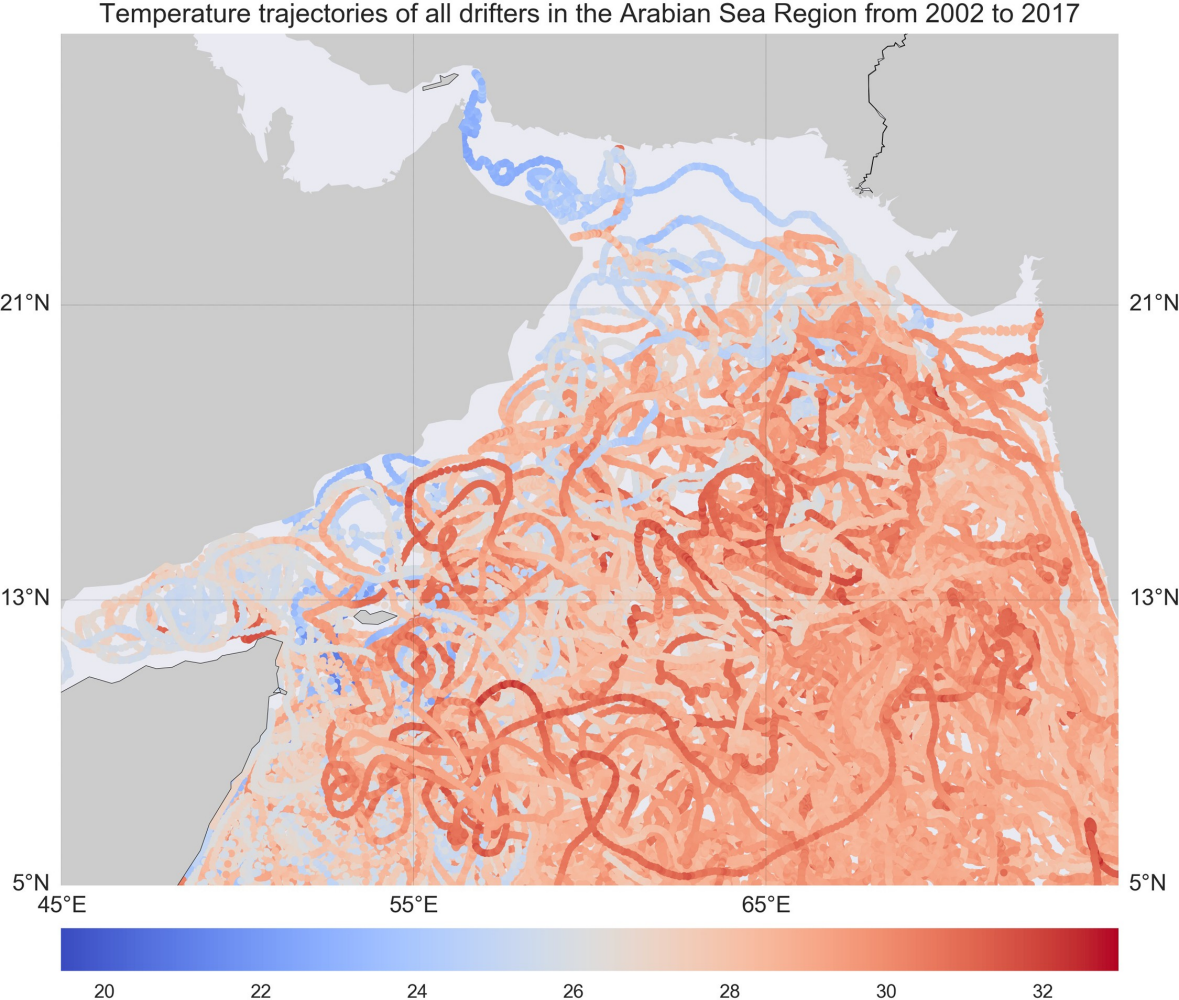
Temperature trajectories of all drifters in the Arabian Sea Region from 2002 to 2017.

The temporal evolution of the satellite images reflects both physical and biological dynamics. To impose the structures of physical drivers (advection) onto the data sample, we utilized the drifter array data from the NOAA’s Global Drifter Program (GDP). These freely drifting buoys provide information about the upper ocean currents that are responsible for the advection of the planktonic particles [48]. The Lagrangian trajectory of each float is retrieved from the database as a time series of variables representing the location, velocity field, and sea surface temperature. We note that each float has a typical lifetime of a couple years and has different launch times. Therefore, the drifter dataset is highly heterogeneous in both time and space. Fig 3 displays the temperature

measurements of all the drifters in the Arabian Sea. It is known that the Lagrangian drifter trajectories are highly chaotic [49–51], and the prediction of particle trajectories has been a challenging research task [52]. There have been recent research results on using Kalman filter and data assimilation with various physical models, namely, the Gauss–Markov Lagrangian particle model [8], the Eulerian velocity field [9, 11], and the upper ocean horizontal momentum balance model from Ekman dynamics [10], to track the position and velocity of the floats. For comparison in our study, we are introducing and imposing statistical structure on the latent state variables to capture the joint dynamics among the *Chl a*, the spatio-temporal information of the floats, namely, the latitude, longitude, velocity, speed and distance to the coast, and the physico-chemical predictors, such as *CDOM*, *KD490*, *T865*, *PAR*, and *SST4*, where *KD490* is the diffuse attenuation coefficient at 490 nm using the Lee algorithm indicating light under the sea surface, and *PAR* is photosynthetically available radiation indicating light on the sea surface. The predictive plots generated by the vLDS model in our study (as displayed in Section “Discussion and Conclusions”) reveal the relationships among the physical and physico-chemical ocean profiles and the *Noctiluca* blooms inside a collection of chaotic drifter trajectories in the Arabian Sea region from 2002 to 2017.

### Combining multiple dataset

We merged the satellite data with the buoy data to generate a Lagrangian dataset. It is a collection of multivariate time series for each drifter with a unique id to combine the information from the satellites, namely, *Chl a*, *CDOM*, *KD490*, *T865*, *PAR*, and *SST4*, and the data associated with the drifters including *id*, *time*, latitude, longitude, velocity components, speed, and distance to the coast. Fourteen features were selected in our experiments; the variance inflation factors (VIF) on multicollinearity of all the predictors are listed in Table 1. The drifter *id* and *time* are mainly used for ordering and grouping data in the vLDS model. The twelve (12) remaining factors represent the physical and physico-chemical variables that is related to the evolution of *Noctiluca* blooms [25–26]. Since the timescale of the phytoplankton reproduction is on the order of a few days [53], we carried out a resampling process to match the frequencies of both datasets to the same level. At the same frequency, we interpolated the variables from the satellite dataset onto the specific spatial and temporal points of the Lagrangian drifter dataset, in order to make each observation in the drifter dataset more informative. This process builds up a multivariate time series for each drifter id with all the physico-chemical and physical information embedded on the drifter trajectory. Furthermore, this interpolation process is repeated for all other features from the satellites.

**Table 1:** Factors used in the vLDS model.

Factor	Description	Median (range)	VIF
*id*	Drifter id	Totally 230 drifters with 5594 data records	—
*time*	Time of the observation	Nov 1 to Mar 31, from 2002 to 2017	—
*lat*	Latitude	12.92 °N (5.03–26.99)	2.16
*lon*	Longitude	63.86 °E (45.07–74.95)	1.62
*ve*	Eastward velocity component	-4.54 cm/s (-122.87–108.47)	1.09
*vn*	Northward velocity component	1.58 cm/s (-132.75–114.55)	1.01
*spd*	Speed of the drifter	22.14 cm/s (0.86–146.58)	1.36
*dist*	Distance to the nearest coast	390.85 km (1.33–1166)	1.32
*chlor_a*	Chlorophyll *a* concentration (*Chl a*)	0.25 $mg\;m^{-3}$ (0.03–44.77)	—
*sst4*	Nighttime sea surface temperature at 4-micron (*SST4*)	27.06 °C (0–30.06)	1.80
*cdm*	Colored dissolved and detrital organic materials (*CDOM*) absorption coefficient at 443 nm	0.02 $m^{-1}$ (0.01–0.81)	2.64
*kd490*	Diffuse attenuation coefficient at 490 nm using the Lee algorithm (*KD490*)	0.07 $m^{-1}$ (0.04–1.36)	2.51
*t865*	Aerosol optical thickness over water (T865)	0.13 (0.02–0.56)	1.04
*par*	Photosynthetically available radiation (*PAR*)	46.18 $Einstein\;m^{-2}\;{day}^{-1}$ (12.20–57.80)	1.08

We note that each float record has information on its coordinates {lat,lon,time}. For convenience, we denote {lat,lon,time} as {x,y,t}, which is almost always not precisely on the grid of the Ocean Color dataset. To resolve this issue, we now describe the multidimensional interpolation operator to map the chlorophyll *a* concentration onto the GDP float dataset. For any float data point with coordinates $\left\{x_{0},\;y_{0},\;t_{0}\right\}$, we identify the coordinate cube or the grid cell in the Ocean Color dataset that contains this point. In particular, this cube has 8 vertices with coordinates generated by the outer product $\left\{x_{nearest},\;x_{furthest}\right\}⨀\left\{y_{nearest},\;y_{furthest}\right\}\;⨀\;\left\{t_{nearest},\;t_{furthest}\right\}$, where the subscripts *nearest* and *next* indicate the nearest and furthest neighbors in the cube for each coordinate, respectively. We further denote the interpolation weight $w_{x}$ for $x_{0}$ by

$$w_{x}=\frac{x_{0}-x_{nearest}}{x_{furthest}-x_{nearest}}$$

Similarly, we define the weights $w_{y}$ and $w_{t}$ for y and t. Using the function f(x,y,t) to represent the chlorophyll *a* concentration at the coordinate {x,y,t}, we write the interpolated chlorophyll *a* concentration at $\left\{x_{0},y_{0},t_{0}\right\}$ as

$$\begin{array}{cc} f\left(x_{0},y_{0},t_{0}\right)= & \left(1-w_{x}\right)\left(1-w_{y}\right)\left(1-w_{t}\right)\cdot f\left(x_{nearest},y_{nearest},t_{nearest}\right)\\ & +\left(1-w_{x}\right)\left(1-w_{y}\right)w_{t}\cdot f\left(x_{nearest},y_{nearest},t_{furthest}\right)\\ & +\left(1-w_{x}\right)w_{y}\left(1-w_{t}\right)\cdot f\left(x_{nearest},y_{furthest},t_{nearest}\right)\\ & +\left(1-w_{x}\right)w_{y}w_{t}\cdot f\left(x_{nearest},y_{furthest},t_{furthest}\right)\\ & +w_{x}\left(1-w_{y}\right)\left(1-w_{t}\right)\cdot f\left(x_{furthest},y_{nearest},t_{nearest}\right)\\ & +w_{x}\left(1-w_{y}\right)w_{t}\cdot f\left(x_{furthest},y_{nearest},t_{furthest}\right)\\ & +w_{x}w_{y}\left(1-w_{t}\right)\cdot f\left(x_{furthest},y_{furthest},t_{nearest}\right)\\ & +w_{x}w_{y}w_{t}\cdot f\left(x_{furthest},y_{furthest},t_{furthest}\right) \end{array}$$

**Fig 4:**
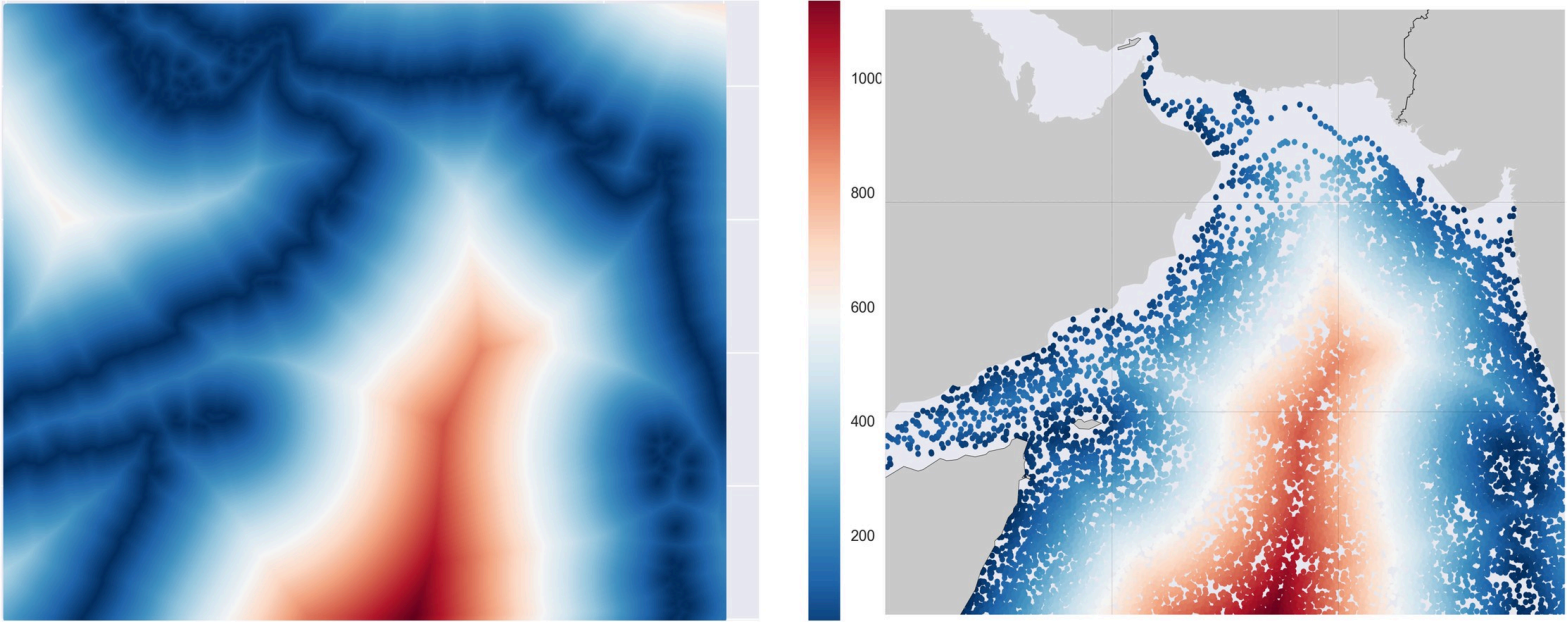
**(A)** Distance to the nearest coast for all the geographical locations in the Arabian Sea Region. **(B)** Interpolated values of the distance to the nearest coast for all the data points in the drifter dataset.

In our implementation, we have applied the interpolation process to the chlorophyll *a* concentration, distance to the nearest coast, and all other predictors. As an illustration of the interpolation process, we display the distance to the nearest coast [40] with a resolution of 4km in Fig 4A and the interpolated distance to the nearest coast for all the floats in Fig 4B.

Using the multidimensional interpolation procedure described above, we map the satellite observations for each of the variables {*‘chlor_a’*, *‘dist’*, *‘cdm’*, *‘kd490’*, *‘t865’*, *‘par’*, *‘sst4’*} onto the GDP floats. Along with the information on the floats, namely {*‘time’*, *‘id’*, *‘lat’*, *‘lon’*, *‘ve’*, *‘vn’*, *‘spd’*}, the interpolated float dataset becomes high dimensional (Table 1). Here *‘chlor_a’* denotes the chlorophyll *a* concentration, *‘dist’* the distance from nearest coast, *‘cdm’* the colored dissolved and detrital organic materials (*CDOM*) absorption coefficient at 443 nm, *‘kd490’* the diffuse attenuation coefficient at 490 nm using the Lee’s algorithm (*KD490*), *‘t865’* the aerosol optical thickness over water (*T865*), *‘par’* the photosynthetically available radiation (*PAR*), ‘*sst4*’ the 4-micron nighttime sea surface temperature (*SST4*), *‘id’* the id of a float, *‘ve’* the eastward velocity component, *‘vn’* the northward velocity component, and *‘spd’* the speed of a float. These physico-chemical and physical factors are chosen to represent all the possible causes for the distribution of the *Noctiluca* blooms in the Arabian Sea. The *‘chlor_a’* measured during the period from November 1 to March 31 are mostly attributed to *Noctiluca* blooms. The ‘*cdm’* measures *CDOM* the amount of dissolved organic materials in the sea water, which supports the growth of the *Noctiluca* [26]. The *‘dist’* measures the distance from the particle to the nearest coast, which is the source of the nutrient rich water. Moreover, in the winter season, the northwestern Arabian Sea off the coast of Oman experiences winter convective mixing from November to January, during which nutrient rich, low-oxygen, cold water is brought to the surface both by convective mixing and by cyclonic eddy activity and benefit the growth of the *Noctiluca* in a complex and nonlinear fashion as described in the “Discussion and Conclusions” Section. The factor ‘*par*’ measures *PAR* the amount of light that is available on the sea surface for the photosynthesis of the symbiotic green algae (Fig 1B) living within *Noctiluca*. The ‘*kd490*’ provides an indication of the transparency of the water column and amount of light that penetrates into the sea water. The ‘*t865’* represents the amount of particles in the atmosphere over the water, an indicator for the atmospheric deposition. The rest of the factors {‘*time’*, *‘id’*, *‘lat’*, *‘lon’*, *‘ve’*, *‘vn’*, *‘spd’*} represent the spatio-temporal information describing the physical transport and dispersal of the *Noctiluca* blooms.

### Data preprocessing for vLDS

Due to the limitation of the satellite coverage mentioned in the “Data Collection” Section, there are missing values in each of the variables in the interpolated dataset. Our focus here is on the chlorophyll *a* concentration *‘chlor_a’*, since it is the key variable for *Noctiluca* blooms. The data structure of the post-processed float dataset is determined by the pre-processing steps for the variable *‘chlor_a’*.

The overall objective of data preprocessing is to keep the microscopic trajectory-based data structure intact, to split the drifter trajectories that are spanning over multiple years, and to remove the drifter trajectories that are too short to be meaningful for the learning algorithm, with due consideration of the physical and physico-chemical meaning. We consider that each period from November 1 to March 31 (during winter monsoon) represents one growth cycle of the *Noctiluca* bloom for a particular float or drifter. For any float with a unique id that has chlorophyll *a* data over two or more cycles, we split the data and assign a new derived float id to the data within each cycle, by adding a small increment 0.05 to the original float id. The resulting dataset allows the vLDS model to learn the trajectory-scale dynamics of each cycle independently.

**Fig 5:**
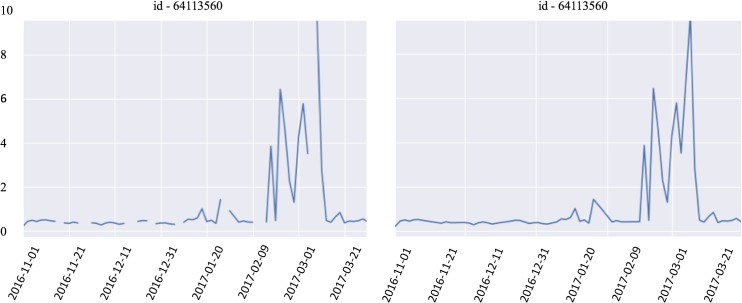
The interpolated time series of ‘*chlor_a’* from the float id 64113560 on the left is shown on the right.

To enhance the quality of the raw input dataset, for each float id, we calculate the percentage of *‘NaN’* values in the dataset in the column *‘chlor_a’* and choose a threshold of 40%. There are two cases. First, if this percentage is smaller than the threshold, the data quality for this particular float is considered to be good, and we interpolate all the missing values for each of the variables in *{‘chlor_a’*, *‘dist’*, *‘cdm’*, *‘kd490’*, *‘t865’*, *‘par’*, *‘sst4’}*. See Fig 5 for an example, in which the time series is interpolated for *‘chlor_a’*. Also, after the interpolation process, there might still be gaps in the time series. For instance, the float might just not have any record, including *‘NaN’*, in a certain short period. In this case, the float will be further split into continuous subseries. Therefore, every interpolated float time series will go through the second step for checking and splitting, which we now describe.

**Fig 6:**
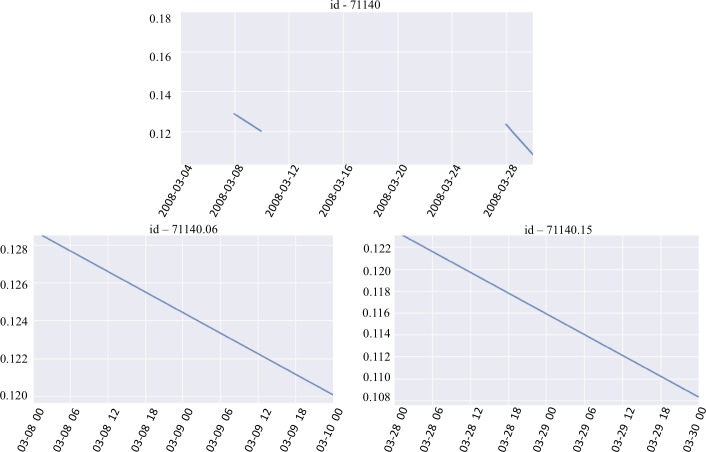
The time series of *‘chlor_a’* from the float 71140 on the top panel is split into 5 different shorter series. Three series of length 1 are dropped, and the remaining two are shown on the bottom panel.

In the other case, if this percentage of *‘NaN’* values is larger than the threshold, the data quality for this particular float is considered unsuitable for interpolation. We split the discontinuous series into smaller continuous series for *‘chlor_a’*. We loop through the time series on *‘chlor_a’* and split it into smaller continuous series for ‘*chlor_a’*. Moreover, we assign a new derived float id to each newly generated shorter series, by adding a small increment 0.03 to the original float id. Also, we drop any series for ‘chlor_a’ of length 1. See Fig 6 for an example, in which we split the time series for ‘chlor_a’ into 5 different shorter series, and we drop three series of length 1. For a threshold of 40%, the irregularity of the drifter dataset is evident from the distribution of the trajectory lengths characterized by [10, 16, 26, 53] at the [20%, 40%, 60%, 80%] quantiles, respectively. For a threshold of 20%, the quantiles are [7, 10, 16, 30]. For a threshold of 10%, the quantiles are [7, 9, 11, 19]. It is evident that with a tighter threshold, such as 20% or 10%, many of the long time series will be split into too many shorter time series. Many of the resulting shortened series are too short, comparing to the time series in the heldout dataset. Therefore, we have fixed a threshold of 40% in the following experiment.

### Linear dynamical systems (LDS)

After preprocessing, the merged GDP floats dataset consisted of 186 float records. For each float, the measurement is a multivariate time series, given by a vector

y:={lat,lon,ve,vn,spd,dist,chlor_a,par,cdm,t865,kd490,sst4}.

**Fig 7:**
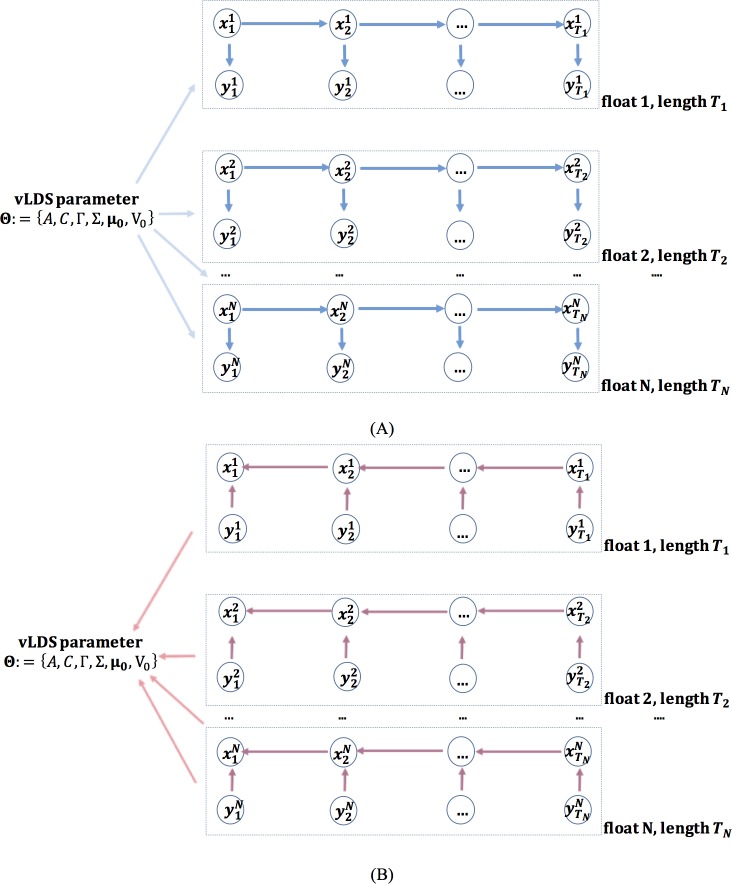
**(A)** Information flow of the forward iteration in the Expectation step of the vLDS model for computing the mean and the variance of the posterior marginal latent variables $x_{i}|\theta_{old},y_{1},y_{2},\ldots y_{i}$ at time i. **(B)** Information flow of the backward iteration in the Expectation step of the vLDS model for computing the mean and the variance of the posterior marginal latent variables $x_{i}|\theta_{old},y_{1},y_{2},\ldots y_{T}$ based on all the information from times 1 to T.

We use **$x^{n}=(x_{1}^{n},\;x_{2}^{n},\ldots x_{T_{n}}^{n})$** to denote the latent variables, $y^{n}=(y_{1}^{n},y_{2}^{n},\ldots y_{T_{n}}^{n})$ the observations from the float n, and $T_{n}$ the length of the time series of the float n. The plain version Linear Dynamical System (LDS) is an adaptive procedure that can learn from data to recover the latent relationship between y and x, using assumptions of linear relationships at time i between $x_{i}$and $x_{i-1},\;y_{i}$ and **$x_{i}$,** with Gaussian Noise. For the moment, we omit the superscript n and focus on one float. More specifically, we assume that for a specific float, the time series of the latent variables and the observations hold the following relationships:

$$\begin{array}{cc} x_{i}=Ax_{i-1}+w_{i} & w_{i}\;\sim \;N(w_{i}|0,\Gamma \;)\\ y_{i}=Cx_{i}+v_{i} & v_{i}\;\sim \;N(v_{i}|0,\Sigma )\\ x_{1}=\mu_{0}+u & u\;\sim \;N\left(u|0,{\text{V}}_{0}\right)\;, \end{array}$$ (1)

where $w_{i},\;v_{i},\;u$ are noise terms. The LDS model fits the model parameters $\Theta :\;=\;\left\{A,C,\Gamma ,\Sigma ,\mu_{0},V_{0}\right\}$ by taking the expectation over the latent variables $x_{i}|\theta_{old}$, and maximizing the log-likelihood of the complete data {x,y|θ}, where $\theta_{old}$ is the model parameter from the previous iteration and θ is the parameter that we are seeking at the current iteration. In the expectation step, with the parameter $\theta_{old}$, the mean and the variance of the posterior marginal latent variables $x_{i}|\theta_{old},y_{1},y_{2},\ldots y_{i}$ at time i (see float 1 in Fig 7A) and the mean and the variance of the posterior marginal latent variable $x_{i}|\theta_{old},y_{1},y_{2},\ldots y_{T}$ based on the information at all time (see float 1 in Fig 7B), are calculated using the forward and backward iterations. Here, T is the total length of a particular time series in a float. These marginal variables lead to the sufficient statistics of the complete data {x,y|θ}, namely the $E_{x|\theta_{old},y_{1},y_{2},\ldots y_{T}}[x_{i}]$, $E_{x|\theta_{old},y_{1},y_{2},\ldots y_{T}}[x_{i}x_{i-1}^{T}]$, $E_{x|\theta_{old},y_{1},y_{2},\ldots y_{T}}[x_{i}x_{i}^{T}]$. Using these sufficient statistics [54, 55], we obtain the updated LDS model parameters $\Theta :\;=\;\left\{A,C,\Gamma ,\Sigma ,\mu_{0},V_{0}\right\}$. The graphical model [56] of the plain LDS is schematically plotted in Fig 7A and 7B as one branch, for instance, the branch of float 1. The workflow of the plain LDS model is described in Algorithm 1.

Algorithm 1. Plain version of training the LDS model on one float

1. initialize $\Theta :\;=\;\left\{A,C,\Gamma ,\Sigma ,\mu_{0},V_{0}\right\}$, *iter = 1*, *maxiter = 100*, $rtol\;=\;10^{-4}$
2. for (*iter* < *maxiter*) do
3.  Expectation step:
4.  forward iteration to compute $x_{i}|\theta ,y_{1},y_{2},\ldots y_{i}$
5.  backward iteration to compute $x_{i}|\theta ,y_{1},y_{2},\ldots y_{T}$
6.  compute $E_{x|\theta ,y_{1},y_{2},\ldots y_{T}}[x_{i}],\;E_{x|\theta ,y_{1},y_{2},\ldots y_{T}}[x_{i}x_{i-1}^{T}],\;E_{x|\theta ,y_{1},y_{2},\ldots y_{T}}[x_{i}x_{i}^{T}],\;llh(iter)$
7.  if $llh\left(iter\right)-llh\left(iter-1\right)\geq rtol*llh(iter-1)$ break; end if
8.  Maximization step:
9.  update $\Theta :\;=\;\left\{A,C,\Gamma ,\Sigma ,\mu_{0},V_{0}\right\}$
10.  end for

### Variable-length linear dynamical systems (vLDS)

In this study, each float generates one or more statistically independent time series of the *Chl a* concentration, due to the interpolation or splitting process discussed in the “Data Preprocessing” Section. For the preprocessed dataset with 186 floats, we treat it as multiple multivariate time series, each with a unique id. Also, we note that the lengths of the time series in the dataset are mostly different, due to the irregularity of the longevity of the floats. The variable-length Linear Dynamical Systems model is specifically designed to address this situation, as it summarizes and recovers the latent dynamics from multiple multivariable time series with a different time span.

To fit the vLDS model, we start with some initial parameter $\Theta_{0}$, which is shared across all floats in the dataset. We keep the superscript n here. The Expectation step is carried out on each float id, using a two-loop forward and backward smoothing step to compute the conditional expectations of the sufficient statistics of the complete data {x,y|θ}, namely the $E_{x|\theta_{old},y_{1},y_{2},\ldots y_{T_{n}}}[x_{i}]$, $E_{x|\theta_{old},y_{1},y_{2},\ldots y_{T_{n}}}[x_{i}x_{i-1}^{T}]$, $E_{x|\theta_{old},y_{1},y_{2},\ldots y_{T_{n}}}[x_{i}x_{i}^{T}]$. In the maximization step, we use the averaging formula derived in Eq (5) across all floats to update the model parameter $\Theta :\;=\;\left\{A,C,\Gamma ,\Sigma ,\mu_{0},V_{0}\right\}$.

We emphasize that for a particular drifter n, the multivariate observation $y_{i}^{n},\;i\;=\;1,2,3,\ldots T_{n}$, contains all the physical and physico-chemical information along a drifter trajectory at time i, and it is the multivariate latent random variable $x_{i}^{n}$ that we are solving for from the vLDS model to represent the hidden dynamics between different components of $y_{i}^{n}$, namely,

{lat,lon,ve,vn,spd,dist,chlor_a,par,cdm,t865,kd490,sst4}.

It is possible that some of the components of $y_{i}^{n}$, for instance, the **t865** in our study, as demonstrated in the “Discussion and Conclusions” Section, are not much involved in the latent dynamics. Therefore, the dimension of the latent space recovered by the latent variable $x_{i}^{n}$ might be smaller than the dimension of the observations $y_{i}^{n}$. In this study, the latent dimension in **$x_{i}^{n}$**, as determined by the cross-validation procedure, turns out to be 11, and the dimension of the observations **$y_{i}^{n}$** is 12.

To test the hypotheses in the “Background” Section, we first generate the predicted values of $\tilde{y_{i}^{n}}$ by using Eq (1) with the recovered latent variables $x_{i}^{n}$ from the vLDS model. Since the vLDS model automatically maximizes the log-likelihood of the complete data, including the latent variable x and the observational variable y, it is beneficial to visualize the predictive plots of all predictors along the drifter trajectories, as shown in the “Discussion and Conclusions” Section, and study the performance metric R-squared, as described in the “Results” Section.

### Probabilistic computation of vLDS

We assume that the observed multivariate variable $y_{i}^{n}$, including the chlorophyll *a* concentration, of each float is evolving independently with other floats, except that they are driven by the same underlying physico-chemical and physical forces. It is this assumption that allows the vLDS model to share the same model parameters $\Theta :\;=\;\left\{A,C,\Gamma ,\Sigma ,\mu_{0},V_{0}\right\}$ in all branches in Fig 7A and 7B and makes the vLDS model a powerful algorithm to summarize and capture the population-level structures along drifter trajectories. Multiple variants of the LDS model have been applied successfully at the microscopic scale in several research areas such as computational neuroscience [19–20, 57–58] and sound tracking [22], in which the authors considered various extensions of LDS to data sequences with fixed length. Our vLDS method is different, and particularly designed for trajectory-based data sequences with variable lengths. The irregularity of the drifter dataset is evident from the distribution of the trajectory lengths characterized by [10, 16, 26, 53] at the [20%, 40%, 60%, 80%] quantiles, respectively

Algorithm 2. Training the vLDS model on the cross-validation dataset with many floats

1. initialize $\Theta :\;=\;\left\{A,C,\Gamma ,\Sigma ,\mu_{0},V_{0}\right\}$, *iter* *=* *1*, *maxiter* *=* *100*, $rtol\;=\;10^{-4}$
2. for (*iter* < *maxiter*) do
3.  Expectation step:
4.  for float n=1,…,N do
5.   forward iteration to compute $x_{i}|\theta ,y_{1},y_{2},\ldots y_{i}$
6.   backward iteration to compute $x_{i}|\theta ,y_{1},y_{2},\ldots y_{T_{n}}$
7.   compute $E_{x|\theta ,y_{1},y_{2},\ldots y_{T_{n}}}[x_{i}]$, $E_{x|\theta ,y_{1},y_{2},\ldots y_{T_{n}}}[x_{i}x_{i-1}^{T}]$, $E_{x|\theta ,y_{1},y_{2},\ldots y_{T_{n}}}[x_{i}x_{i}^{T}]$, llh
8.   sum $llh\left(iter\right)\;=\;llh\left(iter\right)+llh$
9.   end for
10.  if $llh\left(iter\right)-llh\left(iter-1\right)\geq rtol∙llh(iter-1)$ break; end if
11.  Maximization step:
12.  update $\Theta :\;=\;\left\{A,C,\Gamma ,\Sigma ,\mu_{0},V_{0}\right\}$ using Eqs (3)–(5)
13.  end for

Although the distribution of the complete data {x,y|θ} depends on the model parameter θ, we omit the dependence on θ for notational ease in the following derivation. For a particular float n, letting i be the time step and $T_{n}$ be the total length of the time series, the distribution of the complete data (namely the observations y and the latent variables x) can be written as

$$P\left(\left\{x^{n},y^{n}\right\}\right)=P\left(x_{1}^{n}\right)\overset{T_{n}}{\underset{i=2}{\prod }}P(x_{i}^{n}|x_{\;i-1}^{n})\overset{T_{n}}{\underset{i=1}{\prod }}P(y_{i}^{n}|x_{i}^{n})$$

$$\text{log}P\left(\left\{x^{n},y^{n}\right\}\right)=logP\left(x_{1}^{n}\right)+\overset{T_{n}}{\underset{i=2}{\sum }}logP\left(x_{i}^{n}|x_{\;i-1}^{n}\right)+\overset{T_{n}}{\underset{i=1}{\sum }}logP\left(y_{i}^{n}|x_{i}^{n}\right)$$ (2)

$$=-\frac{1}{2}{\left(x_{1}^{n}-\mu_{0}\right)}^{\text{T}}V_{0}^{-1}\left(x_{1}^{n}-\mu_{0}\right)-\frac{1}{2}\;-\overset{T_{n}}{\underset{i=2}{\sum }}\left\{\frac{1}{2}{\left(x_{i}^{n}-Ax_{\;i-1}^{n}\right)}^{\text{T}}\Gamma^{-1}\left(x_{i}^{n}-Ax_{\;i-1}^{n}\right)\right\}\;–\frac{T_{n}-1}{2}\log\left|\Gamma \right|\;-\overset{T_{n}}{\underset{i=1}{\sum }}\left\{\frac{1}{2}{\left(y_{i}^{n}-Cx_{i}^{n}\right)}^{\text{T}}\Sigma^{-1}\left(y_{i}^{n}-Cx_{i}^{n}\right)\right\}\;–\frac{T_{n}}{2}\log\left|\Sigma \right|+const.$$

By the assumption of independence, the joint probability of the observations and state variables across all floats expands into the product of the joint probability of the observations and state variables of all the time series generated by each float n= 1,2…N

$$P\left(\left\{x^{1},\ldots ,x^{N},y^{1},\ldots ,y^{N}\right\}\right)=\overset{N}{\underset{n=1}{\prod }}P\left(x_{1}^{n}\right)\overset{T_{n}}{\underset{i=2}{\prod }}P\left(x_{i}^{n}|x_{\;i-1}^{n}\right)\overset{T_{n}}{\underset{i=1}{\prod }}P\left(y_{i}^{n}|x_{i}^{n}\right)$$ (3)

$$log\;P\left(\left\{x^{1},\ldots ,x^{N},y^{1},\ldots ,y^{N}\right\}\right)=\overset{N}{\underset{n=1}{\sum }}logP\left(\left\{x^{n},y^{n}\right\}\right)$$ (4)

We note that, for each float, the preprocessed data of this float might generate multiple multivariate time series (see the “Data Preprocessing” Section for more details.) Under the traditional i.i.d. assumptions, the objective function of the vLDS model is simply the addition of the objective functions for each individual time series' log-likelihood with all the parameters Θ:=$\left\{A,C,\Gamma ,\Sigma ,{\mu_{0},V}_{0}\right\}$ that define the plain version LDS for each float (or each box-branch in Fig 7) being shared across all the floats. Using the Expectation-Maximizing algorithm, the Expectation step can be carried out using a two loop backward and forward iteration for each time series independently, due to the conditional independence of the state variables across different time series of different floats. However, in the maximization step we need to average the vLDS model parameter across all the time series from all the floats.

The derivation of the update formula for the vLDS model parameters Θ:=$\left\{A,C,\Gamma ,\Sigma ,{\mu_{0},V}_{0}\right\}$ follows directly by taking derivatives of the complete data log-likelihood with respect to each component in Θ and by using the standard results from the Maximum Likelihood Estimators of the mean and variance for the Gaussian Distribution. For a dataset of many floats, the complete data log-likelihood has an addition summation sign running through n=1,2…N in Eq (4). We use $T_{n}$, instead of T, to denote the length of the time series of the float n. Given the fact that the derivative of a linear combination of functions is a linear combination of derivatives of each function, we can write the updating formula for the maximization step as:

$$C^{new}=\left(\overset{N}{\underset{n=1}{\sum }}\overset{T_{n}}{\underset{i=1}{\sum }}y_{i}^{n}E{\left(x_{i}^{n}\right)}^{T}\right){\left(\overset{N}{\underset{n=1}{\sum }}\overset{T_{n}}{\underset{i=1}{\sum }}E\left(x_{i}^{n}{\left(x_{i}^{n}\right)}^{T}\right)\right)}^{-1}$$

$$\Sigma^{new}=\frac{1}{\sum_{n=1}^{N}T_{n}}\left(\begin{array}{c} \overset{N}{\underset{n=1}{\sum }}\overset{T_{n}}{\underset{i=1}{\sum }}{\left(y_{i}^{n}(y_{i}^{n}\right)}^{T}-C^{new}E\left(x_{i}^{n}\right){\left(y_{i}^{n}\right)}^{T}-y_{i}^{n}E{\left(x_{i}^{n}\right)}^{T}C^{new}\\ +C^{new}E(x_{i}^{n}{\left(x_{i}^{n}\right)}^{T})C^{new}\;) \end{array}\right)$$

$$A^{new}=\left(\overset{N}{\underset{n=1}{\sum }}\overset{T_{n}}{\underset{i=2}{\sum }}E\left(x_{i}^{n}{\left(x_{i-1}^{n}\right)}^{T}\right)\right){\left(\overset{N}{\underset{n=1}{\sum }}\overset{T_{n}}{\underset{i=2}{\sum }}E\left(x_{i-1}^{n}{\left(x_{i-1}^{n}\right)}^{T}\right)\right)}^{-1}$$ (5)

$$\Gamma^{new}=\frac{1}{\sum_{n=1}^{N}(T_{n}-1)}\left(\begin{array}{c} \overset{N}{\underset{n=1}{\sum }}\overset{T_{n}}{\underset{i=2}{\sum }}(E(x_{i}^{n}{\left(x_{i}^{n}\right)}^{T}-A^{new}E\left(x_{i-1}^{n}{\left(x_{i}^{n}\right)}^{T}\right)-E\left(x_{i}^{n}{\left(x_{i-1}^{n}\right)}^{T}\right)A^{new}\\ +A^{new}E(x_{i-1}^{n}{\left(x_{i-1}^{n}\right)}^{T}){\left(A^{new}\;\right)}^{T}) \end{array}\right)$$

$$\mu_{0}^{new}=\frac{1}{N}\overset{N}{\underset{n=1}{\sum }}E\left(x_{1}^{n}\right)$$

$$V_{0}^{new}=\frac{1}{N}\overset{N}{\underset{n=1}{\sum }}\left(E\left(x_{1}^{n}{\left(x_{1}^{n}\right)}^{T}\right)-E\left(x_{1}^{n}\right)E\left({\left(x_{1}^{n}\right)}^{T}\right)\right)$$

The workflow of the vLDS model is described in Algorithm 2, and the averaging of the vLDS model parameters Θ across all the floats in Eq (4) is carried out in step 12. During this procedure, the vLDS model adaptively learns the latent dynamics of the underlying process. We have fitted the chlorophyll *a* concentration for the particular float in Fig 5 with id 64113560. The result is displayed in the “Discussion and Conclusions” Section.

Each Expectation-Maximization cycle of the LDS model for Gaussian random variables is guaranteed to increase the value of the complete data log-likelihood. Therefore, a standard stopping criterion for the Expectation-Maximization algorithm is based on the complete data log-likelihood in Eqs (2) and (4) with a relative tolerance $rtol\;=\;10^{-4}$ and maximum iteration 100. (The source code of the vLDS implementation is available at: https://bitbucket.org/yy2250cu/vlds-oceancolormodeling/src/).

One of the key model parameters in the LDS modeling is the dimension k of the latent space, namely, the number of components in the latent variable x. It is the dimension of the subspace generated by the projection of the full feature space onto the latent subspace, whose projection back onto the full feature space in Eq (1) under the vLDS linear transformation matrix C maximizes the complete data log-likelihood. A larger k indicates that there are more independent factors in the latent space of x driving the underlying dynamical system of {x, y}. Moreover, varying the values of the dimensionality k induces a family of different vLDS models (1)—(3) indexed by k. To select the model with the most appropriate parameter k, we carry out a 10-fold cross-validation [59–61] on the parameter k and choose the optimal k that achieves the maximum complete data log-likehood on the test dataset. More specifically, we group the dataset by float ids. We hold a portion of the floats ids and consider them as the heldout testing dataset. We take the rest of the float ids as the cross-validation dataset. In the cross-validation step, we split the cross-validation set evenly into 10 folds. Each time we take one fold as the testing dataset, we take the rest as the training dataset. We fit the vLDS parameter $\Theta :\;=\;\left\{A,C,\Gamma ,\Sigma ,\mu_{0},V_{0}\right\}$ on the training dataset and compute the complete data log-likelihood on the testing dataset using this newly fitted parameter Θ. The complete data log-likelihood is averaged for different testing fold for a fixed k. Then, we repeat the entire process for different values of k. See Table 2 for the complete data log-likelihood generated by different cross-validation trails. The averaged complete-data log-likelihood across different testing sets is maximized at k=11.

**Table 2:** 10-fold cross-validation on the parameter k. The optimal k that achieves the maximum complete data log-likelihood on the test set is k=11. The unit of the test-dataset log-likelihood in the table is $10^{4}$.

	k	1	2	3	4	5	6	7	8	9	10	11	12
fold	$llh_{test}$												
1		-1.44	-1.36	-1.33	-1.23	-1.15	-1.19	-1.15	-1.04	-1.14	-1.18	-1.01	-1.65
2		-1.33	-1.22	-1.20	-1.09	-0.98	-1.02	-0.95	-0.83	-0.94	-0.97	-0.81	-1.42
3		-1.58	-1.53	-1.47	-1.27	-1.25	-1.19	-1.26	-1.10	-1.19	-1.15	-1.02	-1.46
4		-1.29	-1.18	-1.15	-1.04	-0.98	-0.97	-0.94	-0.82	-0.88	-0.86	-0.80	-1.11
5		-1.44	-1.33	-1.30	-1.17	-1.12	-1.11	-1.10	-0.91	-1.06	-1.11	-0.88	-1.57
6		-1.58	-1.45	-1.45	-1.34	-1.22	-1.36	-1.31	-1.17	-1.27	-1.19	-1.14	-1.70
7		-1.71	-1.55	-1.52	-1.34	-1.25	-1.22	-1.19	-1.02	-1.07	-1.09	-0.95	-1.31
8		-1.14	-1.02	-1.02	-0.93	-0.84	-0.88	-0.87	-0.73	-0.76	-0.76	-0.71	-0.95
9		-1.63	-1.48	-1.44	-1.27	-1.17	-1.18	-1.19	-0.99	-1.06	-1.10	-0.94	-1.35
10		-1.52	-1.40	-1.35	-1.22	-1.20	-1.13	-1.11	-0.92	-0.99	-1.04	-0.85	-1.33
Average		-1.47	-1.35	-1.32	-1.19	-1.11	-1.12	-1.11	-0.95	-1.04	-1.05	**-0.92**	-1.38

**Fig 8:**
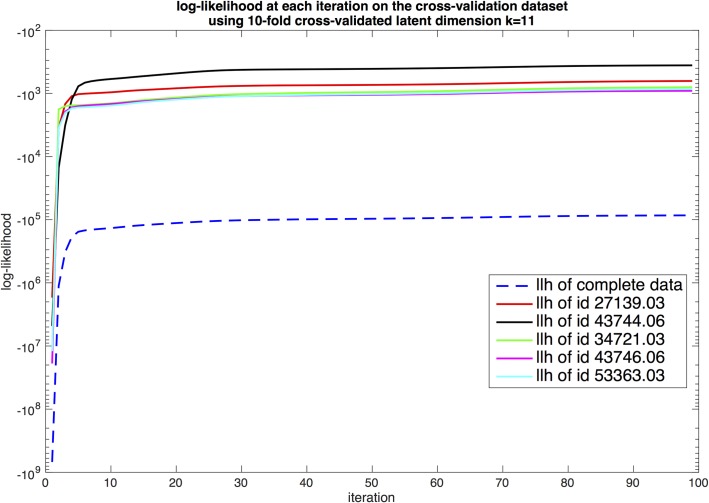
Convergence history of the log-likelihood of the complete cross-validation dataset and a sample of the convergence history for 5 floats.

With the optimal value k=11of the latent space dimension identified, we fit the vLDS model one more time with the full cross-validation dataset to generate the vLDS model parameter. In Fig 8, we display the log-likelihood convergence of the Expectation-Maximization algorithm for the complete cross-validation dataset and five individual floats in the cross-validation dataset. We note that from Eqs (2)–(4), the log-likelihood of the complete cross-validation dataset is the sum of the log-likelihood of each individual floats in the cross-validation dataset (step 8 in Algorithm 2.)

## Results

**Fig 9:**
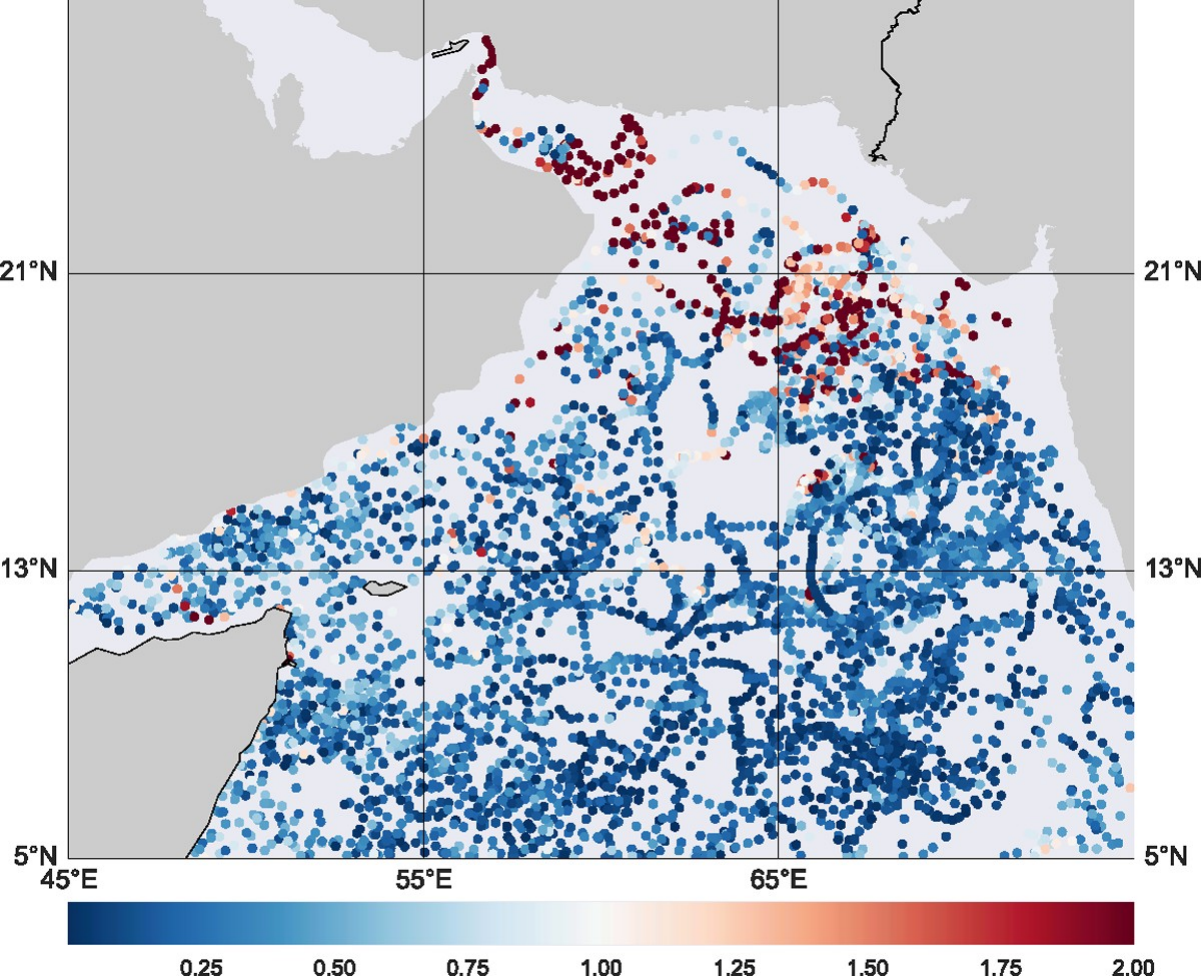
Spatial distribution of the vLDS prediction error for the chlorophyll *a* concentration (*chlor_a*).

**Fig 10:**
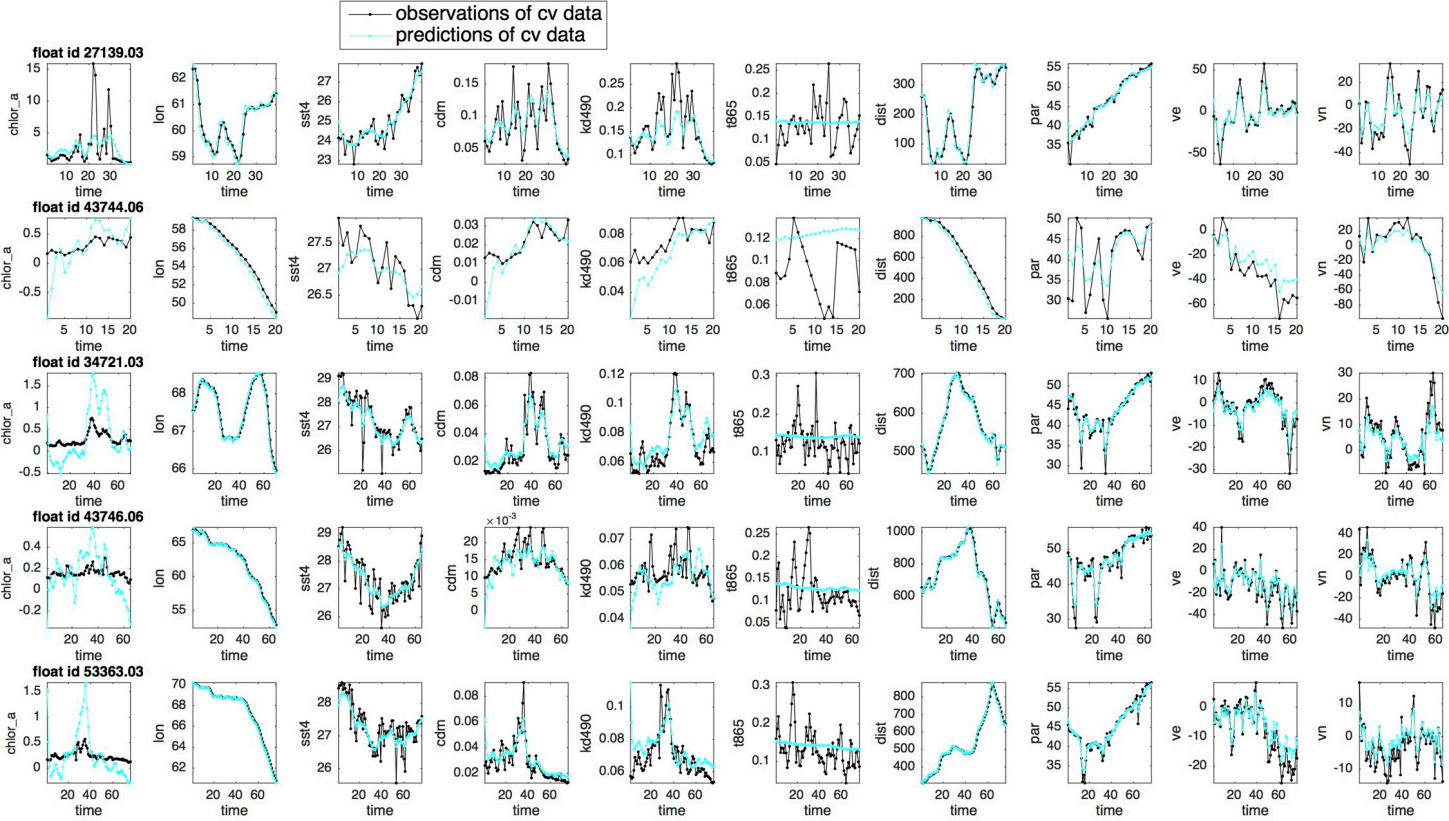
Predictions of the drifter profiles for the floats in the cross-validation dataset, using the expected conditional mean of the latent variables at the last iteration of the Expectation-Maximization algorithm.

**Fig 11:**
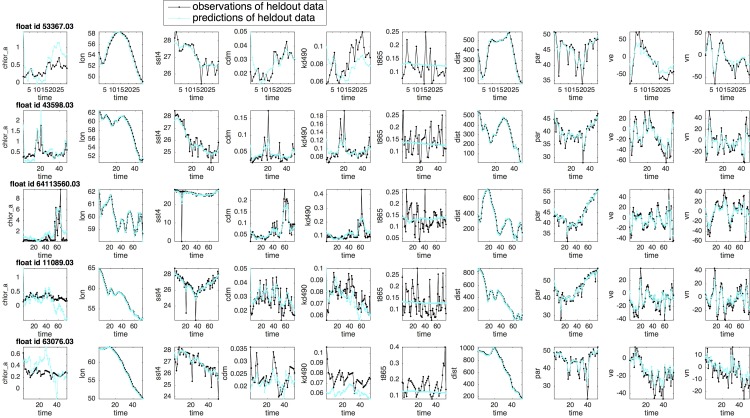
Predictions of the drifter profiles for the floats in the heldout testing dataset, using the expected conditional mean of the latent variables generated by one iteration of the forward-backward smoothing process of the Expectation-Maximization algorithm with the vLDS model parameter $\Theta :\;=\;\left\{A,C,\Gamma ,\Sigma ,\mu_{0},V_{0}\right\}$ optimized on the cross-validated dataset.

With the optimally selected latent space dimension k=11, the vLDS algorithm obtains a set of model parameters $\Theta :\;=\;\left\{A,C,\Gamma ,\Sigma ,\mu_{0},V_{0}\right\}$ when the stopping criterion inside the Expectation-Maximization algorithm is reached. The spatial distribution of the vLDS prediction error for the chlorophyll *a* concentration is shown in Fig 9. Fig 10 shows the prediction results for some drifter ids in the cross-validation dataset, using Eq (1) and the expected conditional mean of the latent variables at the last iteration of the Expectation steps 4, 5, and 6 in Algorithm 2. The dark lines are the observations, and the cyan lines are the predictions. Most of the hidden dynamics of the float profiles inside the cross-validation dataset are well captured by the vLDS model. The $R^{2}$ values of the drifters in Fig 10 are 0.95, 0.98, 0.98, 0.98, 0.99, respectively. We note the positive correlations among *‘chlor_a’*, *‘cdm’*, and *‘kd490’* in the recovered vLDS latent dynamics (cyan lines in Fig 10) at the local drifter-scale and population-level in the cross-validation dataset. The model captures this correlation with some overshooting or undershooting in certain regions. Also, *‘t865’*, the aerosol optical thickness over water, turns out to be independent of the chlorophyll *a* concentration and other ocean profiles. Moreover, the spatial information, namely, the longitude, latitude, velocity, speed of the float, and distance to the nearest coast, is all well recovered by the vLDS model (*lat* and *spd* are not shown in Figs 10 and 11 due to space limitations.)

We next examine on the robustness of the vLDS model. The floats in the heldout testing dataset are not used in the cross-validation process or the model’s parameter estimation process. Therefore, the heldout testing dataset is totally unknown to the vLDS learning algorithm. We use the cross-validated latent dimension k=11, and the model parameter $\Theta :\;=\;\left\{A,C,\Gamma ,\Sigma ,\mu_{0},V_{0}\right\}$ generated by training the vLDS model on the cross-validation dataset. Applying one iteration of the forward-backward smoothing process, namely, i.e., one iteration of the Expectation steps 4, 5, and 6 in Algorithm 2, to each float in the heldout testing dataset, we obtain the predictions of their profiles (Fig 11). Most of the hidden dynamics along drifter trajectories for the floats in the heldout testing dataset, which is totally unknown to the learning algorithm, is well captured by the vLDS model. The $R^{2}$ values of the drifters in Fig 11 are 0.93, 0.97, 0.98, 0.99, 0.99, respectively. They clearly demonstrate the generalization ability of the vLDS model’s capability to summarize and capture the local population-level structures along the drifter trajectories on unknown datasets.

We note again the positive correlations among *‘chlor_a*, *‘cdm’*, and *‘kd490’* in the recovered vLDS latent dynamics (cyan lines in Fig 11) at the local population-level for the drifters in the heldout dataset. Even in this heldout testing dataset, the model captures this correlation to a large degree, with some overshooting or undershooting in certain regions. Again, *‘t865’*, the aerosol optical thickness over water, seems to be independent of the *Chl a* concentration and other ocean profiles in the heldout dataset. The vLDS model simply estimates the mean value for the variable ‘t865’ in both the cross-validation and heldout datasets. However, by comparing the predictions generated by the vLDS model for ‘t865’ and for other variables, we conclude that the variable ‘*t865*’ is not much involved in the latent dynamics of the *Noctiluca*’s growth. Otherwise, the predicted values of ‘*t865*’ should match its observations in Figs 10 and 11, and the $R^{2}$ of ‘*t865*’ in Table 3 should not be too small. Evidently, the vLDS model indicates that there is no strong relationship between ‘*t865*’ and the latent dynamics of the *Noctiluca*’s growth at the population-level along the drifter trajectories, a feature lacking in models that do not take the local trajectory-based population-level structure into consideration. Moreover, the spatial information of the heldout floats, namely, longitude, latitude, velocity, speed of the float, and distance to the nearest coast, is all well recovered by the vLDS model.

**Table 3:** R-squared $\left(R^{2}\right)$ metric for the cross-validation dataset and heldout testing dataset. $R^{2}$ is computed for each individual feature and aggregated together for all features.

		cross-validation data					heldout testing data			
	SSTotal	SSE	SSE(stdev)	$R^{2}$	$R^{2}$(stdev)	SSTotal	SSE	SSE(stdev)	$R^{2}$	$R^{2}$(stdev)
*lat*	1.17E+5	3.78E+2	2.45E+1	0.99	2.08E-4	4.32E+3	1.86E+1	2.14E+0	0.99	4.94E-4
*lon*	2.32E+5	4.42E+2	2.58E+1	0.99	1.13E-4	3.52E+3	3.23E+1	2.31E+0	0.99	6.56E-4
*ve*	2.37E+6	3.67E+5	2.02E+4	0.85	8.52E-3	1.51E+5	2.49E+4	3.72E+3	0.84	2.46E-2
*vn*	2.36E+6	4.51E+5	2.70E+4	0.81	1.09E-2	1.07E+5	1.63E+4	1.47E+3	0.85	1.37E-2
*spd*	1.78E+6	2.62E+5	1.49E+4	0.85	8.38E-3	7.20E+4	1.09E+4	8.77E+2	0.85	1.22E-2
*dist*	3.55E+8	2.05E+6	1.14E+5	0.99	3.42E-4	1.78E+7	1.52E+5	9.24E+3	0.99	5.19E-4
*cdm*	1.65E+1	1.88E+0	4.91E-1	0.89	3.01E-2	3.40E-1	4.00E-2	1.03E-2	0.87	2.91E-2
*kd490*	2.43E+1	8.02E+0	2.31E+0	0.67	9.51E-2	3.19E-1	1.39E-1	3.96E-2	0.56	1.24E-1
*t865*	1.34E+1	1.29E+1	5.91E-1	0.04	4.51E-2	7.30E-1	7.49E-1	7.28E-2	-0.04	9.97E-2
*par*	2.41E+5	1.80E+4	1.09E+3	0.93	4.55E-3	9.43E+3	7.87E+2	7.35E+1	0.91	7.81E-3
*sst4*	1.20E+4	2.07E+3	4.25E+2	0.83	3.51E-2	1.02E+3	5.13E+2	2.06E+2	0.49	2.02E-1
*chlor_a*	4.60E+4	2.19E+4	4.28E+3	0.52	9.31E-2	2.59E+2	1.46E+2	5.47E+1	0.43	2.11E-1
Aggreg.	3.62E+8	3.17E+6	1.82E+5	0.99	5.03E-4	1.82E+7	2.06E+5	1.57E+4	0.98	8.62E-4

In addition to the above-mentioned correlations that are recovered correctly from the vLDS predictive data stream $\left\{\tilde{y_{i}^{}}\right\}$, it is evident that both spatio-temporal {*time*, *lon*, *lat*, *dist*}, physical {*ve*, *vn*, *spd*}, and physico-chemical factors {*sst4*, *cdm*, *kd490*, *par*} are involved in this 11-dimensional (k=11) latent dynamics for the *Noctiluca* blooms. It is important to note that vLDS serves as a mechanism to exclude irrelevant variables, such as *‘t865*’, for the underlying microscopic latent dynamics at the local drifter-scale, which is the *Noctiluca*’s growth in this study.

To quantify the performance of the vLDS model, we use the R-squared metric $\left(R^{2}\right)$. In Table 3, the total sum of squares (SSTotal), sum of squared errors of predictions (SSE), and $R^{2}$, which is the portion of the variance captured by the predictive model, are computed for both the cross-validation and heldout testing datasets. The standard deviations (stdev) of SSE and $R^{2}$are also listed in Table 3. Although the vLDS model has the log-likelihood of the complete data in Eqs (3) and (4) as its own performance metric, we use $R^{2}$ here for an intuitive interpretation. The quantitative results reflect the visualization in Figs 10 and 11. The feature ‘*t865*’ has a very small value in its $R^{2}$ metric and does not exhibit any predictive power. The vLDS recovers the spatio-temporal information well, and explains most of the variance in the physico-chemical factors {‘*cdm*’, ‘*kd490*’, ‘*par*’, ‘*sst4*’, ‘*chlor_a*’}.

## Discussion and conclusions

We have introduced a new model vLDS and showed that it offers a new local-scale trajectory-based data analysis tool to recover biogeochemical mechanisms underlying chaotic drifter trajectories that might be unobservable at the macroscopic scale or accessible only in controlled laboratory experiments. The vLDS model generates predictions that recover the causal relationship among the *Noctiluca* blooms, physical dispersal, and physico-chemical environments (Figs 10 and 11 and Table 3.) The model’s generalization capability also summarizes, recovers, and predicts the latent dynamics from unknown heldout testing datasets, thus inspiring confidence in our local-scale findings along drifter trajectories and macroscopic findings of pooled data. The highly correlated relationships between the *‘chlor_a’* and *‘cdm’* (colored dissolved organic matter *CDOM*), and between the *‘chlor_a’* and ‘*kd490’* (light under the sea surface) are close to linear. The tightly correlated relationships between the *‘chlor_a’* and *‘par’* (light on the sea surface *PAR*), and between the *‘chlor_a’* and *‘sst4’* (sea surface temperature *SST4)* are nonlinear. The vLDS model does not provide evidence of a strong relationship between ‘*t865*’ and the latent dynamics of the *Noctiluca*’s growth.

Furthermore, in the vLDS model, individual components are not assumed to be mutually independent in the multivariate random variable. After the prediction step, the linear correlations are only one aspect of insights that can be obtained from vLDS. In fact, correlations are linear relationships. The latent dynamics recovered by vLDS predictions, on the other hand, is not simply a linear correlation. Although the vLDS is named Linear, it is evident from the updating formula Eq (1) that recursively applying linear transformations on the latent variable $x_{i-1}$ makes both the latent data stream $\left\{x_{i}\right\}$ and the predictive data stream $\left\{\tilde{y_{i}^{}}\right\}$ generated by the vLDS highly nonlinear. So the data stream $\left\{\tilde{y_{i}^{}}\right\}$ recovered or generated by vLDS is not simply linear correlated. Instead, it is on a low-dimensional nonlinear manifold generated by vLDS. It is evident that both spatio-temporal {*time*, *lon*, *lat*, *dist*}, physical {*ve*, *vn*, *spd*}, and physico-chemical factors {*sst4*, *cdm*, *kd490*, *par*} are involved and correctly recovered in this 11-dimensional (k=11) latent dynamics for the *Noctiluca* blooms. It is important to observe that vLDS also serves as a mechanism to exclude irrelevant variables, such as *‘t865*’, for the underlying local trajectory-scale latent dynamics in general, beyond the results in this study for *Noctiluca*’s growth.

These results confirm the macroscopic hypotheses in the “Background” Section from the local trajectory-scale perspective, and confirms the impact of both the physical transport and physico-chemical factors of light and nutrients, proxies for the latter being *CDOM*, on the distribution of the *Noctiluca* blooms. Also, the test results imply that the nutrient and light (light on and under the sea surface) are important positive factors for the *Noctiluca*’s growth. Regarding the atmospheric deposition ‘t865’, the vLDS model does not provide evidence of a strong relationship between ‘t865’ and the latent dynamics of the *Noctiluca*’s growth (Figs 10 and 11 and Table 3). Due to the fact that the nutrient dynamics involving the atmospheric deposition may have lagging and cumulating effects, further research regarding the role of the atmospheric deposition in the *Noctiluca*’s growth is needed. It has also been confirmed from the drifter dynamics recovered by the vLDS Model that *Noctiluca* grow faster in lighted than in dark areas on the sea surface and in the sea water.

We have demonstrated the effectiveness of the vLDS model as a local-scale trajectory-based statistical modeling tool for detecting important causal relationships in biogeochemical processes. Although the trajectories of the oceanographic probing devices are chaotic and the dataset is high dimensional, the vLDS model is very parsimonious on model parameters. The model only requires $\Theta :\;=\;\left\{A,C,\Gamma ,\Sigma ,\mu_{0},V_{0}\right\}$ and the latent-space dimension k to be able to summarize all the drifters in the Arabian Sea region from 2002 to 2017. The predictive dynamics matches the local-scale observations along drifter trajectories well, and affords tremendous confidence in support of the macroscopic hypotheses.

Furthermore, the intertwined relationships recovered by the vLDS model between the physical and physico-chemical dynamics of the *Noctiluca* blooms and the intertwined relationships among the physico-chemical factors such as ‘*cdm*’ and ‘*kd490*’ have inspired us to use inference tools to quantify the isolated impact of the physico-chemical factors that are responsible for the *Noctiluca* blooms as ‘*chlor_a*’ in the Arabian Sea region. The vLDS model presented here is fully generalizable to other datasets for other applications, such as larval transport in marine ecology.

## Code availability

The source code for the variable-length Linear Dynamical System (vLDS) method is available at: https://bitbucket.org/yy2250cu/vlds-oceancolormodeling/src/

## Supporting information

S1 Software Source code of the vLDS implementation. (DOCX)

S1 Appendix Previous research on Noctiluca blooms. (DOCX)
