# Supplementary material for: Robust learning algorithms for capturing oceanic dynamics and transport of Noctiluca blooms using linear dynamical models
Source: PLoS One. 2019 Jun 13;14(6):e0218183. doi: 10.1371/journal.pone.0218183 (PMC6564007; doi:10.1371/journal.pone.0218183)
Supplement: S1 Software — (DOCX) [file pone.0218183.s001.docx]

**Supporting information**

**Article title: Robust Algorithms for Capturing Oceanic Dynamics and Transport using Linear Dynamical Systems with Latent Variables**

**Authors: Yan Yan, Tony Jebara, Ryan Abernathey, Joaquim Goes, Helga Gomes**

**The following Supporting Information is available for this article:**

**S1 Software. Source code of the vLDS implementation.**

**S1 Software. Source code of the vLDS implementation.**

The source code of implementing the variable-length Linear Dynamical System (vLDS) method is available at:

https://bitbucket.org/yy2250cu/vlds-oceancolormodeling/src/
