# Supplementary material for: Robust learning algorithms for capturing oceanic dynamics and transport of Noctiluca blooms using linear dynamical models
Source: PLoS One. 2019 Jun 13;14(6):e0218183. doi: 10.1371/journal.pone.0218183 (PMC6564007; doi:10.1371/journal.pone.0218183)
Supplement: S1 Appendix — (DOCX) [file pone.0218183.s002.docx]

**Supporting information**

**Article title: Robust Algorithms for Capturing Oceanic Dynamics and Transport using Linear Dynamical Systems with Latent Variables**

**Authors: Yan Yan, Tony Jebara, Ryan Abernathey, Joaquim Goes, Helga Gomes**

**The following Supporting Information is available for this article:**

**S1 Appendix. Previous research on Noctiluca blooms.**

**S1 Appendix. Previous research on Noctiluca blooms.**

Traditionally, photosynthetic diatoms supported the Arabian Sea food chain; zooplankton preyed primarily on diatoms, which were in turn grazed by fish. Since early 2000s, the ecosystem of the Arabian Sea appears to have changed [27], with large and widespread blooms of *Noctiluca* (mixotrophic dinoflagellate *Noctiluca* *scintillans*) superseding diatoms which dominated winter monsoon blooms. The authors in [26] were able to discover that the annual outbreaks of *Noctiluca* were linked to the up shoaling of hypoxic waters from depth into the euphotic zone. Within a decade and half, *Noctiluca* blooms have virtually replaced diatoms at the base of the food chain, marking what appears to be an unprecedented ecosystem shift.

In previous studies, the authors in [25-26], [28] relied on satellite observations and in-situ data sampling and biologically controlled experiments on board of research vessels, to describe the underlying dynamics governing the transport, the growth and decay of the *Noctiluca* blooms in the Arabian Sea region. It has been demonstrated that *Noctiluca* is capable of migrating up and down in the water column depending on conditions at the surface. Like all dinoflagellates [29-32], a stable water column is essential for the growth and proliferation of *Noctiluca* as large surface blooms. Since it is a mixotroph, it can meet its metabolic requirements via feeding on external prey or through photosynthesis by thousands of green “endosymbionts,” living within its central symbiosome (Fig 1B). This flexibility gives it an edge on diatoms, which survive on sunlight alone. When feeding on an external source of organic matter Noctiluca tends to accumulate significant amounts of ammonia [33] and lipids [27], making them highly buoyant, allowing them to accumulate at the sea surface for prolonged periods of time. When present at the surface *Noctiluca* can be easily transported by physical oceanographic processes, where there appear in satellite ocean color in association with micro and meso-scale eddies, filaments and streamers. Over the past decade in particular, *Noctiluca* blooms have become more intense and widespread. The blooms of 2015 and 2017 were the largest on record occupying an area almost thrice the size of the State of Texas.

Summarizing this complex biogeochemical process, recent research [26-27] provided indications that the biological or environmental trigger for the *Noctiluca* blooms were nutrient rich, low-oxygen waters, brought to the surface by an annually recurring mesoscale cyclonic eddies. The north-eastward propagation of these eddies and filaments associated with them appeared to be responsible for the gradual spread of the bloom from their inception in the Sea of Oman in Dec. towards the east by January until mid-March (Fig 1C). The underlying forces responsible for the dispersal of the winter-time *Noctiluca* blooms are multiple (Dec. towards the east by January until mid-March). While *Noctiluca's* unique physiological properties play an important role in maintaining the blooms at the surface and the overall integrity of the bloom, its dispersal is primarily due to ocean currents and the directions in which they move and mix with other water masses [34-39].

Due to the temperature gradient across the land mass and ocean, the winter monsoon from November to February is cold and dry and is blowing from the Indian subcontinent. Moreover, the winter monsoon cools the surface of the ocean and induce convective mixing that brings nutrients from depth to the surface. Nutrients waters brought to the surface by winter convective mixing during the winter monsoon foster large *Noctiluca* blooms of the Arabian Sea [26]. This mixing at meso- and sub-mesoscale occurs and changes over a seasonal basis, due to the interactions between the ocean and atmosphere that are driven by the monsoonal wind forcing [40-41].
